# Prevalence of low-body cell mass evaluated by bioelectrical impedance vector analysis related to aging

**DOI:** 10.1590/1806-9282.20240088

**Published:** 2024-11-11

**Authors:** Luis García-Castañeda, Lilia Castillo-Martinez, Víctor Manuel Mendoza-Núñez, Guadalupe Silvia García De La Torre, Wendy Daniella Rodríguez-García

**Affiliations:** 1 Instituto Nacional de Ciencias Médicas y Nutrición Salvador Zubirán, Departamento de Nutrición Clínica – Mexico City, México.; 2 Universidad Nacional Autónoma de México, Facultad de Estudios Superiores Zaragoza, Unidad de Investigación en Gerontología – Mexico City, México.; 3 Universidad Nacional Autónoma de Mexico, Facultad de Medicina, Departamento de Salud Pública – Mexico City, México.; 4 Universidad Nacional Autónoma de México, Facultad de Estudios Superiores Zaragoza, Licenciatura en Nutriología – Mexico City, México.

**Keywords:** Prevalence, Cell mass, Aging, Hydration, Electric impedance, Community dwelling

## Abstract

**OBJECTIVE:**

The objective of this study was to determine the prevalence of low-body cell mass by sex and age in a community-dwelling population.

**METHODS:**

In this retrospective study, 981 community-dwelling adults aged ≥35 years (648 women and 333 men) from Mexico City were recruited in a subway station between February and April 2012. Demographic data, anthropometry, and body composition were assessed, and self-reported comorbidities were recorded in the consulting room. Impedance values were obtained using mono-frequency equipment at 50 kHz. For the diagnosis of low-body cell mass by bioelectrical impedance vectorial analysis: resistance (R, Ohm) and reactance (Xc, Ohm) values were adjusted for height to obtain impedance vector (R/H and Xc/H) and then plotted in the R/Xc to perform the bioelectrical impedance vectorial analysis RXc Z-score analysis.

**RESULTS:**

The total prevalence of low body cell mass by bioelectrical impedance vectorial analysis was 29.4% (n=288) with a 95%CI 26.5–32.3%; was higher in men (39%) compared with women (24.4%) (p<0.0001). The group aged>75 years had the highest prevalence (men: 85.3%, p<0.001; women: 63.3%, p<0.001). The bioelectrical impedance vectorial analysis RXc Z-score analysis showed lower cell mass in men aged>75 years (R Z-score −0.30, Xc Z-score −2.13), and women aged 65–74 years also presented with overhydration (Z-score −2.6, Z-score −1.27).

**CONCLUSION:**

The prevalence of low body cell mass, evaluated by bioelectrical impedance vectorial analysis, increased with age. RXc Z-score analysis could be a useful tool to evaluate nutritional status and changes in hydration in community-dwelling populations.

## INTRODUCTION

In health-related clinical practice, body composition evaluation, weight, and body mass index (BMI) are commonly used. However, a person classified with normal nutritional status could hide muscle depletion and low body cell mass related to lifestyle (inactivity and inadequate diet) or metabolic and neuroendocrine changes associated with aging or pathological conditions^1,2^. Body cell mass (BCM) can be defined as fat-free mass without bone mineral mass or extracellular water. BCM is the sum of the work-, oxygen-, glucose-, and potassium-rich cellular components of the human body. Therefore, it is the most metabolically active human body compartment involved in O_2_ consumption, CO_2_ production, and energy expenditure, and its main fractions are the cellular components of the muscles and viscera. It comprises tissues that are most likely to be affected by physical activity, nutrition, or disease^3^. The assessment and quantification of BCM are particularly important because they provide a greater physiological and functional reserve that protects against mortality and morbidity^4^.

The bioelectrical impedance (BIA) method used to evaluate body composition provides direct, point-of-care measurements that allow non-invasive assessment of an individual's soft tissue hydration and cell membrane integrity. BIA considers the human body as a network of resistors, including physiological fluids, intracellular fluids, and extracellular fluids^5^. The dielectric properties of cell membranes are related to their area and integrity. The integrity of cell membranes is a determinant of membrane potential, and together with area, is probably a determinant of cell function^4^. Reactance (Xc) provides an index of cell population density (e.g., cells in water) and may be interpreted as a semiquantitative index of cell mass and a biomarker of cell integrity or quality^4^. Reactance (Xc) may therefore also reﬂect body cell mass.

In addition, the bioelectrical impedance vectorial analysis (BIVA) approach allows us to obtain simultaneous changes in tissue hydration or soft-tissue mass, independent of regression equations, or body weight. Therefore, BIVA can be interpreted accurately, even if patients are at extremes of weight, volume, or altered hydration^6^. BIVA considers two axes in the resistance (R) and reactance (Xc) graphs, with the major axis indicating the state of hydration and the minor axis referring to corporal tissues. The individuals located within the ellipses of 50 and 75% reported a normal body composition in tissue and hydration, whereas those located outside the 75% ellipses represented alterations in body composition^7^. In addition, the statistical conversion of R and Xc was divided by height to Z-scores. The Z-score is the number of standard deviations from the mean value of the reference group^6^. Z-scores can provide information about an individual's measured score relative to others in the distribution and enable researchers to compare the body composition of different study populations. Piccoli et al. used this method to compare BIVA data for a variety of disease groups^8^.

Empirical evidence from experiments involving the cooking of vegetable and meat specimens demonstrates major reductions in reactance (Xc) and phase angle (PhA), which corresponds to the histological evidence of cell membrane destruction^9^.

During aging, it has been demonstrated that the presence of chronic disease accelerates the loss of muscle mass and functionality, which are both components of body cell mass^10^. However, there are no studies on Mexican community-based subjects regarding the frequency of low body cell mass. This study aimed to determine the prevalence of low body cell mass using BIVA by sex and age group in a community-dwelling population.

## METHODS

This retrospective study included community-dwelling adults from Mexico City. This study was performed in the subway of Mexico City (from February to April 2012) and approved by the Ethics Committee of the Universidad Nacional Autónoma de México Zaragoza Campus, which approved the research protocol for this study (PAPIIT IN308411-2). The inclusion criteria were subjects older than 35 years, apparently healthy or with controlled comorbidities by self-report, and born in Mexico who agreed to participate in the study. The exclusion criteria were pregnant women, high-performance athletes, athletes with any traumatic event or prosthesis in the upper or lower extremities, hospitalizations in the last 6 months (positive history of inflammatory or neurologic diseases), and artificial joints or osteosynthesis plates performed by orthopedic surgery. Each participant was informed of the aims of the study and consented to participate actively. The measurements were performed in the consulting room of a station, which is a commuting transportation system used by a large percentage of the population living in the city (1,623,828,642 users in 2015); therefore, it was considered an adequate place for the sample. Age, sex, and comorbidities (diabetes and hypertension) were also self-reported.

The sample size was calculated using the formula for estimating prevalence, considering a 95% confidence level, and the desired margin of error was 0.05, with an assumed prevalence of low muscle mass from a previous study (27%)^10^. The minimum estimated sample size was 303, multiplied by the four sex strata, which was 606. The strategy used for recruitment was to cover a minimum of 30 subjects in four age ranges for each sex using consecutive sampling.

### Anthropometry

Weight and height were measured according to the manual for anthropometric standardization^11^. All subjects were barefoot, avoiding extra weight. The BMI was calculated by dividing the total body weight (kg) by the squared height (m).

### Body composition

Using a standard technique^12^, resistance (R, ohm), reactance (Xc, ohm), and phase angle were measured using whole-body tetrapolar mono-frequency impedance equipment (50 kHz, Quantum X, RJL System). The patient is in a supine position with arms separated from the trunk by approximately 30° and legs separated by approximately 45°, free of drafts and portable electric heaters. Electrodes were placed on the hand, and the foot was unilateral.

For the diagnosis of a low-body mass cell by BIVA, resistance and reactance values were divided by height in meters (R/H, ohm/m and Xc/H, ohm/m) to obtain the impedance vector and then plotted in the R/Xc graph obtained from a healthy Mexican population^13,14^. The RXc-score graph represents the bivariate distribution of the standard deviations of R/H and Xc/H (both with zero mean and unit standard deviation). Bivariate Z-scores were calculated from the mean age group as Z(R)=(R/H mean value of the age group - R/H mean value of the reference population/standard deviation of the reference population) and Z(Xc)=(Xc/H mean value of the age group - Xc/H mean value of the reference population/standard deviation of the reference population). Based on data from the Piccoli et al. study, the BIVA RXc Z-score graph was divided into four quadrants to classify the body composition of populations within 75 and 95% tolerance ellipses. These quadrants were high cell mass, top left; low cell mass, bottom right; edema, bottom left; and dehydration, top right. All vectors were obtained using the BIVA Software 2002^15^.

Subjects with vectors out of the 75% percentile of the tolerance ellipses in the lower right quadrant were classified as having low body cell mass by BIVA. Overhydration was identified when the subject's vector fell below the pole parallel to the major axis of the tolerance ellipses of the 75-tolerance ellipse.

### Statistical analysis

Kolmogorov-Smirnov tested the normal distribution of the continuous variables. For descriptive analysis, variables with normal distribution were reported as mean (standard deviation) and non-normally distributed variables as median (interquartile range). Categorical variables were presented as absolute and relative frequencies. The chi-square test was used for comparisons between categorical variables, and one-way analysis of variance was used to find statistical differences in continuous variables between sex and age groups with Tukey's post hoc comparison. The age- and sex-specific prevalence (%) of low body cell mass was determined by BIVA. Statistical significance was set at p<0.05. All analyses were performed using SPSS version 21.0.

## RESULTS

Recruitment included 1,202 people, of whom 221 (18.3%) were excluded from the final analysis because they presented erroneous data or did not meet the inclusion criteria, such as age. A total of 981 participants were included: 648 women (66%) and 333 men (34%) aged 35–89 years. Table 1 presents the anthropometric and body composition characteristics as well as comorbidities according to sex and age group. Significant differences were found in the variables according to age group (weight, height, R/h, Xc/H, phase angle, low-BCM, altered hydration, and comorbidities) in both men and women.

**Table 1:** Comparison of anthropometric, impedance, and comorbidities variables between age groups according to sex.

Variables	Men					Women				
	Age groups in years									
	35–49 (n=94)	50–64 (n=133)	65–74 (n=72)	>75 (n=34)	p	35–49 (n=102)	50–64 (n=251)	65–74 (n=216)	>75 (n=79)	p
Weight (kg)	78 (13)	77 (14)	71 (9)	69 (11)	<0.001*	66 (10)	68 (13)	64 (11)	60 (11)	<0.001*
Height (m)	1.67 (0.1)	1.65 (0.1)	1.62 (0.1)	1.60 (0.04)	<0.001	1.55 (0.1)	1.51 (0.05)	1.48 (0.05)	1.47 (0.1)	<0.001
BMI (kg/m^2^)	28 (4)	28 (4)	27 (3)	27 (4)	<0.23	27 (4)	30 (5)	29 (5)	28 (5)	<0.001^#^
R/H (ohm/m)	287 (33)	289 (37)	296 (37)	323 (50)	<0.001¥	369 (42)	368 (52)	296 (37)	391 (53)	<0.001¥
Xc/H (ohm/m)	33 (4)	31 (4)	30 (4)	28 (4)	<0.001¶	38 (4)	35 (5)	34 (5)	32 (5)	<0.001¶
Phase angle (°)	6.7 (0.8)	6.2 (0.7)	5.8 (0.8)	4.9 (0.6)	<0.001§	5.9 (0.5)	5.6 (1)	5.2 (0.6)	4.7 (0.8)	<0.001§
Low-BCM, n (%)	16 (17)	47 (35.3)	38 (53.5)	29 (85.3)	<0.001	3 (2.9)	40 (15.9)	65 (30)	50 (63.3)	<0.001
Altered hydration, n (%)	9 (9.6)	28 (21.1)	23 (32.4)	18 (52.9)	<0.001	1 (1)	13 (5.2)	29 (13.4)	22 (27.8)	<0.001
Diabetes, n (%)	2 (2)	15 (12)	9 (18)	7 (25)	<0.001	0	39 (19)	39 (31)	21 (30)	<0.001
Hypertension, n (%)	5 (5)	26 (20)	8 (16)	11 (39)	<0.001	9 (9)	63 (31)	53 (42)	34 (49)	<0.001

Notes: BMI: body mass index; R/H: resistance/height; Xc/H: reactance/height; BCM: body cell mass. Values are presented in mean (SD) or n (%). ANOVA with Tukey post hoc test was used to compare the age groups.

* p<0.05: group 35–49 versus 65–74 and >75; 50–64 versus 65–74 and >75, in men. Group 35–49 versus >75; 50–64 versus 65–74, and >75, in women. ^#^p<0.05: group 35–49 versus 50–64 and 65–74; 50–64 versus >75, in women. ¥p<0.05: group 35–49 versus >75, 50–64 versus >75, 65–74 versus >75 in men. Group 35–49 versus >75, and 50–64 versus >75, in women. ¶p<0.05: group 35–49 versus 50–64, 65–74, and >75; 50–64 versus 65–74 and >75; 65–74 versus >75, in men. Group 35–49 versus 50–64, 65–74, and >75, 50–64 versus >75, 65–74 versus >75, in women. §p<0.05: group 35–49 versus 50–64, 65–74, and >75; 50–64 versus 65–74 and >75; 65–74 versus >75, in men. Group 35–49 versus 65–74 and >75, 50–64 versus 65–74 and >75, 65–74 versus >75, in women.

According to the BIVA, the prevalence of low body cell mass in the study population was 29.4% [95% confidence interval (CI) 26.5–32.3% (n=288)]. Specifically, the prevalence of low body cell mass was higher (p<0.0001) in men [39% (n=130); 95%CI 34.3–44.6%] than in women [24.4% (n=158); 95%CI 21–27.5%]. The highest prevalence (p<0.001) was observed in men and women older than 75 years (85.3 and 63.3%, respectively) compared to all groups (35–49, 50–64, and 65–74) (Table 1).

**Figure 1:**
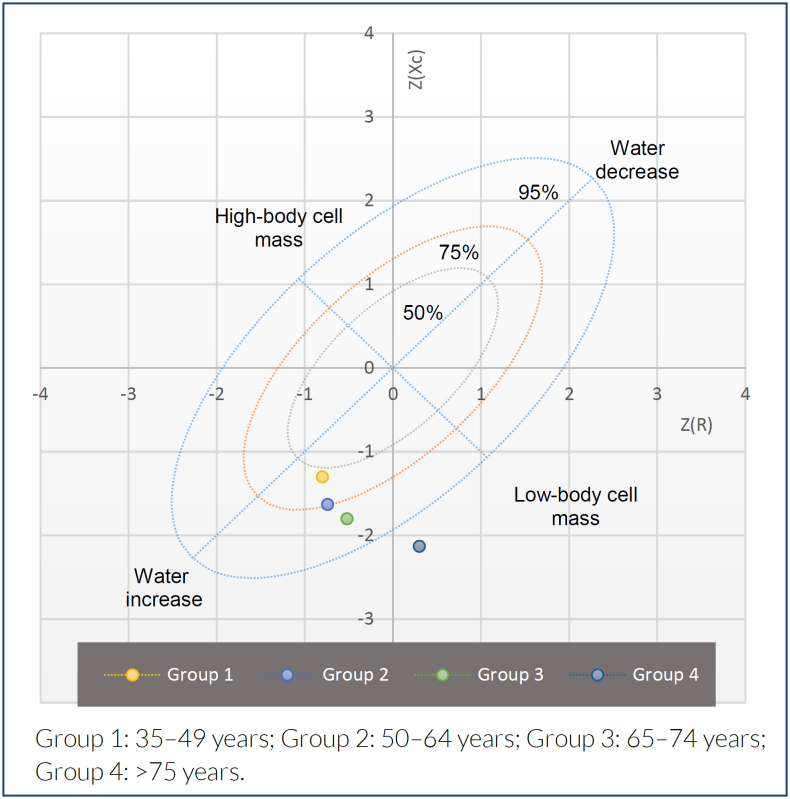
RXc Z-score graph analysis of bioelectrical impedance vector analysis in males of the study population.

**Figure 2:**
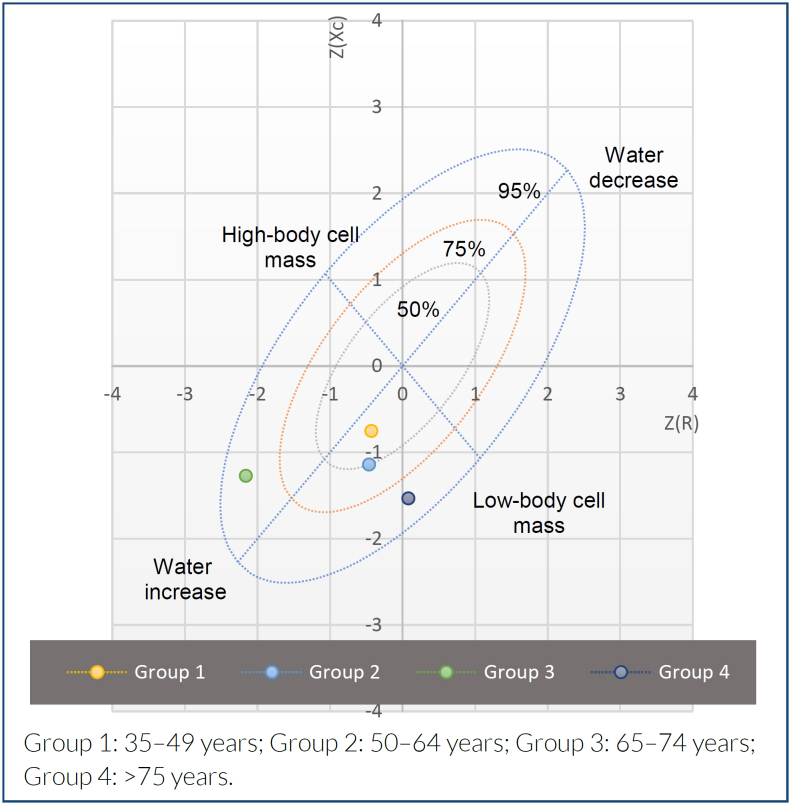
RXc Z-score graph analysis of bioelectrical impedance vector analysis in females of the study population.

The BIVA RXc Z-score analysis is presented in Figures 1 and 2 and shows comparatively lower cell mass in men and women older than 65 years; however, women aged 65 to 74 years also present overhydration.

## DISCUSSION

The main finding of this study was that the prevalence of low body cell mass increased with age in both sexes. Likewise, the results showed that 29.4% of the participants in the study were classified as having low body cell mass, and according to BMI, subjects in all age groups were overweight or obese, failing to detect an altered nutritional state^2^. To the best of our knowledge, this is the first study to report this prevalence and present the difference between sex and age using the BIVA Z-score in community-dwelling adults.

The RXc Z-score graph could be a useful tool in large-scale surveys to evaluate the risk of sarcopenia and subclinical congestion, as well as monitoring changes in hydration and nutritional status in primary care intervention to ensure that changes are in body cell mass and no other body composition compartments. Although body mass index is useful in primary care, in persons older than 35 years, it would be necessary to evaluate metabolically active tissues, and as we can see in women older than 65 years, subclinical body fluid alterations could be indicators of the presence of asymptomatic diseases such as heart, hepatic, or renal failures. Body cell mass has been reported to correlate more specifically with changes in nutritional status^16^.

At present, there have been no studies that evaluated low-body cell mass with vectorial analysis and Z-score in the community persons, to compare the results of this study, because previous research evaluates BCM with prediction equations without considering many pathological states present, especially those influencing nutritional status affecting the proportion between intra- and extracellular body water and one of the assumptions for the use of these equations of constant fat-free mass hydration.

The phase angle is considered an index of physiological status and is associated with hydration. The phase angle reflects the geometric relationship between the resistance and reactance, which includes contributions from both the fluid status (volume and distribution) and cell mass. The phase angle is a surrogate for fluid distribution, specifically the extracellular water (ECW) to intracellular water ratio. Changes in the phase angle can indicate shifts in the fluid balance, such as fluid gain in the ECW or dehydration. Therefore, phase angle can be used as a biomarker to assess the hydration status of individuals^5,17^.

The ﬁndings of this study have implications for clinical practice, evaluation, and detection of low body cell mass, and abnormal ﬂuid distribution can be targeted for treatment with interventions and public health policies, such as resistance exercise. With an emphasis on the population older than 35 years, not only elderly subjects, the impact is reduced.

In clinical practice, Wu et al. proposed the use of the ratio of ECW and body cell mass in the assessment of fluid status in patients with acute kidney injury requiring kidney replacement therapy and its association with an increased risk of mortality. Based on their findings, the authors suggested that physicians should promote the maintenance of BCM and not simply pursue a reduction in overhydration and ECW^18,19^.

This study had some limitations. First, we recruited a convenience sample size at the station of the metro collective transportation system; however, this population comes from all political delegations in the city. Second, we did not use the standard gold method to evaluate body composition; instead, we used BIVA, which is a portable and accurate method to determine low body cell mass considering the state of hydration, besides being independent of regression equations or body weight. In addition, there was a small sample size in groups of subjects older than 75 years because, in this age group, subjects with controlled comorbidities or free hospitalization were scarce.

In conclusion, we identified a high prevalence of low body cell mass evaluated using BIVA in a Mexican community-dwelling population aged ≥35 years.

## ETHICAL STANDARDS

The research protocol was approved by the Ethics Committee of the Universidad Nacional Autónoma de México Zaragoza Campus.
